# TusA influences Fe-S cluster assembly and iron homeostasis in *E. coli* by reducing the translation efficiency of Fur

**DOI:** 10.1128/spectrum.00556-24

**Published:** 2024-06-25

**Authors:** Paolo Olivieri, Arkadiuz Zupok, Tugba Yildiz, Jonathan Oltmanns, Angelika Lehmann, Ewelina Sokolowska, Aleksandra Skirycz, Volker Schünemann, Silke Leimkühler

**Affiliations:** 1 Institute of Biochemistry and Biology, Department of Molecular Enzymology, University of Potsdam, Potsdam, Germany; 2 Department of Physics, University of Kaiserslautern-Landau, Kaiserslautern, Germany; 3 Max-Planck-Institute of Molecular Plant Physiology, Potsdam-Golm, Germany; Forschungszentrum Jülich, Juelich, Germany

**Keywords:** iron uptake, sulfur, iron metabolism, Fe-S, tRNA modification, translation

## Abstract

All sulfur transfer pathways have generally a l-cysteine desulfurase as an initial sulfur-mobilizing enzyme in common, which serves as a sulfur donor for the biosynthesis of numerous sulfur-containing biomolecules in the cell. In *Escherichia coli*, the housekeeping l-cysteine desulfurase IscS has several interaction partners, which bind at different sites of the protein. So far, the interaction sites of IscU, Fdx, CyaY, and IscX involved in iron-sulfur (Fe-S) cluster assembly have been mapped, in addition to TusA, which is required for molybdenum cofactor biosynthesis and mnm^5^s^2^U34 tRNA modifications, and ThiI, which is involved in thiamine biosynthesis and s^4^U8 tRNA modifications. Previous studies predicted that the sulfur acceptor proteins bind to IscS one at a time. *E. coli* TusA has, however, been suggested to be involved in Fe-S cluster assembly, as fewer Fe-S clusters were detected in a ***∆****tusA* mutant. The basis for this reduction in Fe-S cluster content is unknown. In this work, we investigated the role of TusA in iron-sulfur cluster assembly and iron homeostasis. We show that the absence of TusA reduces the translation of *fur*, thereby leading to pleiotropic cellular effects, which we dissect in detail in this study.

**IMPORTANCE:**

Iron-sulfur clusters are evolutionarily ancient prosthetic groups. The ferric uptake regulator plays a major role in controlling the expression of iron homeostasis genes in bacteria. We show that a *∆tusA* mutant is impaired in the assembly of Fe-S clusters and accumulates iron. TusA, therefore, reduces *fur* mRNA translation leading to pleiotropic cellular effects.

## INTRODUCTION

In *Escherichia coli*, the ISC (iron-sulfur cluster) system represents the main Fe-S cluster assembly machinery and is defined as the “*housekeeping*” system for the biosynthesis of Fe-S clusters and other cofactors under normal cellular conditions (1, 2). The ISC system is encoded by genes organized in the *isc* operon, *iscRSUA-hscBA-fdx-iscX* (3). Deletion mutants of *isc* genes show a dramatic reduction of important Fe-S cluster-containing enzymes that are involved in bacterial growth and metabolic pathways (4).

The first protein encoded by the *isc* operon is IscR, identified as a repressor of the *isc* operon itself. IscR binds a [2Fe-2S] cluster (holo-IscR) and represses *isc* operon transcription by binding to the *iscR* promotor (5, 6).

*E. coli* IscS is a homodimeric PLP-dependent L-cysteine desulfurase that generates a persulfide sulfur from L-cysteine, which is converted to L-alanine in the reaction (7, 8). Once the persulfide sulfur is bound to the active-site cysteine of IscS (Cys328), it is transferred to the *scaffold* protein IscU upon which the Fe-S cluster is assembled (8, 9). The persulfide sulfur (S^0^) on IscS must be reduced by ferredoxin to sulfide (S^2−^) to coordinate the iron and form the Fe-S cluster. Despite the iron donor for cluster assembly being unknown, different proteins have been suggested to fulfill this function, including CyaY, IscX, and IscA (10–14). Further CyaY and IscX both have been proposed to be the iron donors based on their ability to bind iron (15–17, 18), despite CyaY not being encoded in the *isc* operon. However, a ternary complex of IscS-IscU-CyaY or IscS-IscU-IscX does not have similar activity as IscS-IscU in the formation of Fe-S clusters (13, 19, 20, 21).

In addition to the Fe-S cluster assembly proteins IscU, Fdx, CyaY, and IscX, several partner proteins have been shown to interact with IscS, including TusA and ThiI (22, 23) Overall, there is a complex protein–protein interaction network involving IscS, which is the master enzyme in the initial mobilization of sulfur from l-cysteine and is responsible for transferring the sulfur to specific sulfur-acceptor proteins (24). So far, the interaction sites of IscU, ThiI, TusA, IscX, Fdx, and CyaY have been mapped on IscS 22, 25, 26) Previous studies predicted that the sulfur acceptor proteins bind to IscS only one at a time (19).

Bacterial cells can survive and adapt to hostile environments and still be able to produce Fe-S clusters, even under oxidative stress and iron-limiting conditions (27). In *E. coli*, this task is fulfilled by the SUF system. The SUF machinery comprises six proteins, the genes for which are organized in the *suf* operon, *sufABCDSE*. While the ISC machinery represents the housekeeping system for Fe-S cluster assembly, the SUF system is mainly used by *E. coli* during oxidative and iron stress conditions. Expression of the *suf* operon is regulated by different transcription factors, including IscR, OxyR, and Fur (28). These proteins control the induction or inhibition of SUF enzyme synthesis, ensuring that an adequate level of Fe-S cluster production occurs during stress conditions (27, 29).

Overall, TusA has a dual role in the cell, delivering sulfur both for the thiomodification of thionucleosides in tRNA and for molybdenum cofactor (Moco) biosynthesis (24). So far, detailed studies have shown that a deletion of *tusA* causes a pleiotropic effect on several additional cellular pathways in *E. coli*, including the enhanced susceptibility of viral infection inhibition by programmed ribosomal frameshifting (30). These pleiotropic effects of a deletion in *tusA* were suggested to be caused by changes in the Fe-S cluster concentration in the cell, thereby revealing a pervading role for Fe-S cluster assembly in the cell (31). Studies showed that elevated levels of TusA in *E. coli* decreased the level of Fe-S clusters. Consequently, when Fe-S clusters become limiting, Fe-S-containing proteins such as MoaA are inactive, which directly results in a decreased activity of molybdoenzymes. On the other hand, overexpression of IscU also reduces the level of active molybdoenzymes in *E. coli* (31). This observation suggests that elevated complex formation of IscU with IscS limits IscS availability for interaction with other proteins, such as TusA. Overall, this emphasizes that the sulfur transfer pathways to sulfur-containing biomolecules are strongly interconnected and likely regulated at the cellular level by the availability of their acceptor proteins. Here, we present evidence in support of this proposal, whereby a *∆tusA* mutant is impaired in the assembly of Fe-S clusters and accumulates iron.

An important protein in the regulation of iron homeostasis in *E. coli* is Fur. Fur is a homodimer with a conserved N-terminal domain in each monomer, which is important for DNA binding, and with a conserved C-terminal dimerization domain (32). Fur has three conserved cysteine residues capable of binding, *in vitro*, not only the physiologically relevant Fe^2+^ cation but also other divalent ions like Mn^2+^, Co^2+^, Cd^2+^, and Cu^2+^. The binding of these metal ions can also activate the DNA binding activity of Fur. The main targets of Fur are the genes involved in iron metabolism. When Fur binds ferrous iron (Fe^2+^-Fur), it interacts with DNA to repress the expression of genes encoding siderophore biosynthesis (like *ent* operon), ferrisiderophore transporters (*fep*, *fhu*, and *fec* genes), energy transducing systems (*circA*, *exbB*, and *exbD*), ferrous iron transporters (*feoABC*), and regulatory factors (like *ryhB*) (33). Fe^2+^-Fur also results in decreased levels of Mn-SOD (superoxide dismutase), encoded by the *sodA* gene, and can down-regulate the expression of its own promotor.

On the other hand, Fe^2+^-Fur can also activate the expression of some genes that encode iron storage proteins, like *ftnA*. This results in a reduced uptake of iron from the external environment, followed by increased iron storage.

The proposed main mechanism of Fur action is as a repressor, in which Fur acts as an iron(II) sensor. Under iron-limiting conditions, insufficient levels of ferrous iron are present, and therefore, Fur is mostly in its apo-form. Apo-Fur is inactive and not able to bind the promotor region of its target genes, which consequently are expressed.

Another mechanism of iron sensing involves indirect gene activation mediated by the regulatory sRNA, RyhB (34–36). RyhB is a small RNA of 90 nucleotides identified in *E. coli* in the early 2000s after the observation that, in a *fur* deletion mutant, the expression of many iron-responsive genes were repressed. The main targets of RyhB are mRNAs transcribed from genes involved in the expression of operons encoding iron-utilizing enzymes, like iron superoxide dismutase, succinate dehydrogenase, and the ISC system. The *ryhB* gene is under the negative control of the active Fe^2+^-Fur and, in turn, can destabilize *fur* mRNA. Transcription of *fur* and translation of its mRNA are inhibited by *ryhB*-Hfq, while transcription is enhanced by OxyR under oxidative stress conditions.

In this study, we analyzed the role of TusA in general Fe-S cluster assembly, as well as in Moco biosynthesis, in further detail. We investigated the role of TusA on the activity of the Fe-S-containing enzymes and extended the studies to the cellular iron and Fe-S cluster levels. We show that a *∆tusA* mutant is impaired in the assembly of Fe-S clusters and accumulates iron. The pleiotropic phenotype of a *tusA* mutant highlights the role of this versatile protein and its importance for cellular sulfur distribution.

## RESULTS

### Whole-cell Mössbauer spectroscopy of BW25113 and ∆*tusA* strains

Previous results showed that in a **∆***tusA* mutant, Fe-S cluster-containing proteins have reduced activities, an impairment that could be rescued by the expression of the *sufABCDSE* operon in these strains (37). To determine the nature of the Fe-S clusters that are reduced in this strain, whole-cell Mössbauer spectroscopy (2) was applied. Advances have been made in applying this technique to whole cells to study differences in the distribution of the overall cellular Fe content and especially toward characterizing the predominant type of Fe-S cluster bound by proteins (2, 38).

**Fig 1:**
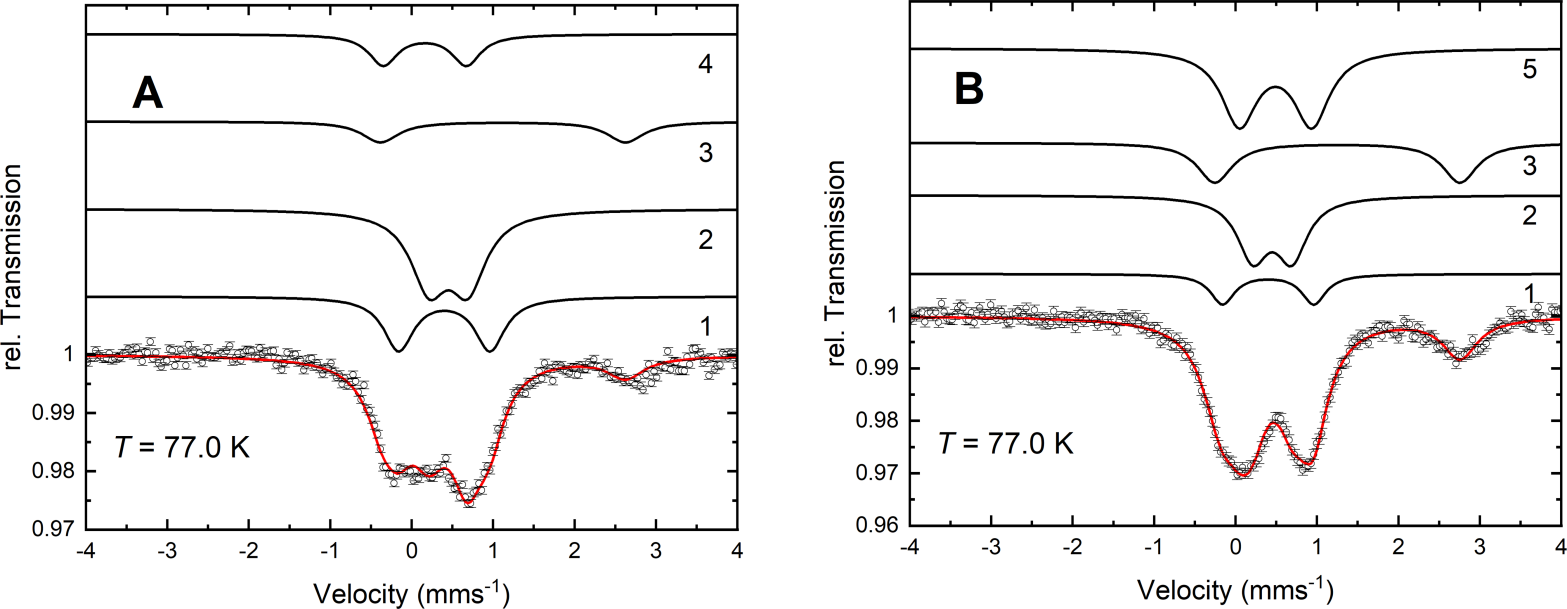
Mössbauer spectra of BW25113 (**A**) and ∆*tusA* strains (**B**) obtained at 77 K. The solid lines represent Lorentzian doublets with the parameters given in Table 1. The relative spectral contributions of the subspectra 1–5 are given in Fig. 2.

To analyze the iron content of cells grown under conditions when molybdoenzymes are expressed, we compared whole cells of the strains BW25113 and its isogenic ∆*tusA* mutant grown anaerobically for 8 h in the presence of 100 µM ^57^Fe-labeled ferric ammonium citrate and 15 mM potassium nitrate. Cells were harvested in the stationary phase and were immediately flash-frozen for Mössbauer spectroscopy. The Mössbauer spectrum of the strain BW25113 obtained at 77 K (Fig. 1A) was analyzed by means of four quadrupole doublets with parameters given in Table 1. Component 1 exhibits an isomer shift of *δ* = 0.40 mm s^−1^ and a quadrupole splitting of Δ*E*_Q_ = 1.12 mm s^−1^. These parameters are characteristic for [4Fe-4S]^2+^ clusters (38) but are also in the range observed for low-spin (S = 0, LS) iron(II) cytochromes (39) and corresponding heme models (2). By comparison, component 2 has an isomer shift of *δ* = 0.45 mm s^−1^ and a quadrupole splitting of Δ*E*_Q_ = 0.48 mm s^−1^. The low value of the quadrupole splitting points to a spherical electron shell observed for high spin (S = 5/2, HS) iron(III) with all five 3d orbitals singly occupied. A mononuclear high-spin iron(III) species with similar parameters (*δ* = 0.5 mm s^−1^ and Δ*E*_Q_ = 0.45 mm s^−1^) has been observed in hyperthermophilic anaerobic *Pyrococcus furiosus* cells (40). Comparable parameters (*δ* = 0.48 mm s^−1^; Δ*E*_Q_ = 0.57 mm s^−1^) have also been reported for iron(III) phosphate oxyhydroxide nanoparticles in mitochondria of Jurkat cells (40). Component 3 has *δ* = 1.25 mm s^−1^ and Δ*E*_Q_ = 3.01 mm s^−1^ and originates from high-spin (S = 2) iron(II) coordinated with 6N/O ligands (2). Component 4 has a rather low isomer shift of *δ* = 0.16 mm s^−1^ and Δ*E*_Q_ = 1.02 mm s^−1^, suggesting a diamagnetic ferrous low-spin species of unknown origin.

**TABLE 1:** Mössbauer parameters of the subspectra 1–5 shown in Fig. 1^*a*^

Component	1 [4Fe‑4S]^2+^, LS Fe^II^ heme		2 HS Fe^III^, nanoparticles		3 HS Fe^II^		4 LS Fe^II^		5 HS Fe^III^, ferritin	
*δ* (mm s^−1^)	0.40	(2)	0.45	(2)	1.25	(2)	0.16	(2)	0.47	(2)
Δ*E*_Q_ (mm s^−1^)	1.12	(2)	0.48	(2)	3.01	(2)	1.02	(2)	0.72	(2)

^*a*^ The relative contributions of the components are shown in Fig. 2. The linewidths for the BW25113 strains (Fig. 1A) are *Γ*_1_ = 0.45 (1), *Γ*_2_ = 0.52 (1), *Γ*_3_ = 0.49 (1), and *Γ*_4_ = 0.33 (1) mm s^−1^. The linewidths for ∆*tusA* strains (Fig. 1B) are *Γ*_1_ = 0.36 (1), *Γ*_2_ = 0.46 (1), *Γ*_3_ = 0.52 (1), and *Γ*_5_ = 0.52 (1) mm s^−1^.

**Fig 2:**
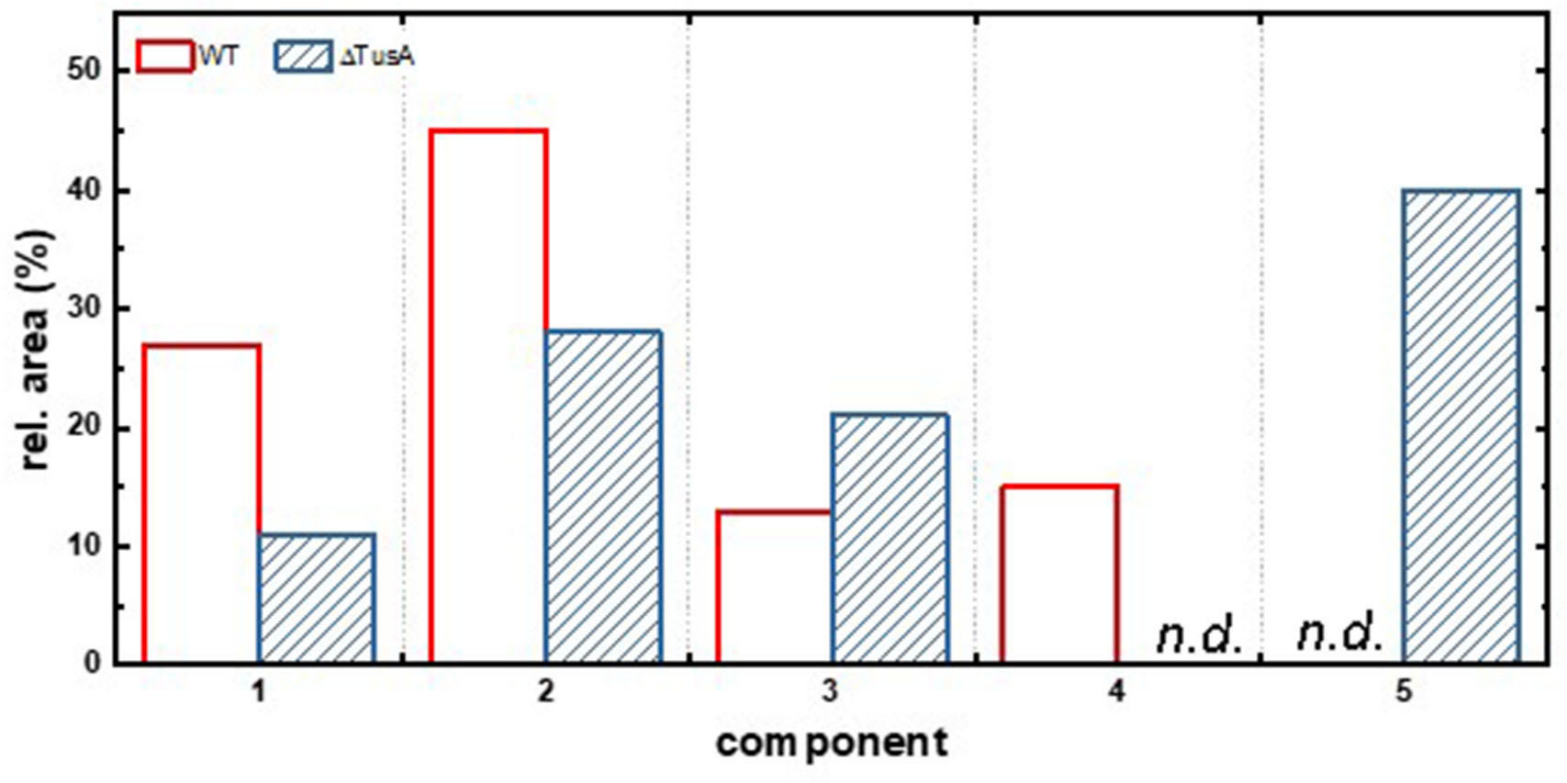
Relative spectral area of components 1–5. Relative spectral area of components 1–5 as obtained from the analysis of the Mössbauer spectra shown in Fig. 1 with the parameters given in Table 1. n.d., not detectable.

The Mössbauer spectrum of cells of the ***∆****tusA* strain (Fig. 1B) shows significant differences. A new component 5 with *δ* = 0.47 mm s^−1^ and Δ*E*_Q_ = 0.72 mm s^−1^ and a relative contribution of 40% evolves at the expense of components 1, 2, and 4, the latter not being present in the ***∆****tusA* strain (Fig. 2). Very similar parameters (*δ* = 0.50 mm s^−1^; Δ*E*_Q_ = 0.75 mm s^−1^) have been attributed to ferritin species in Jurkat cells (40), but we also cannot exclude the presence of nonspecifically bound ferric high-spin species in the ***∆****tusA* strain represented by component 5. It is also worth noting that the nonheme, high-spin iron(II) component 3 almost doubles from 13% in the wild-type sample to 21% in the ***∆****tusA* strain.

We have also compared the spectral area of the Mössbauer spectrum shown in Fig. 1 with that in Fig. 2; this area is proportional to the amount of ^57^Fe species in the sample. The total spectral area decreases from 4.7 arb. units in the native BW25113 strain to 4.1 arb. units in the ∆*tusA* strain, indicating that ^57^Fe levels were lower in the mutant.

In conclusion, the Mössbauer spectra indicate an increase in intracellular free iron and/or ferritin levels and a decrease in [4Fe-4S] cluster levels in the **∆***tusA* mutant compared with the wild type.

### Analysis of the overall iron concentration in cell extracts of strains BW25113, ∆*tusA*, and ∆*mnnA*

**Fig 3:**
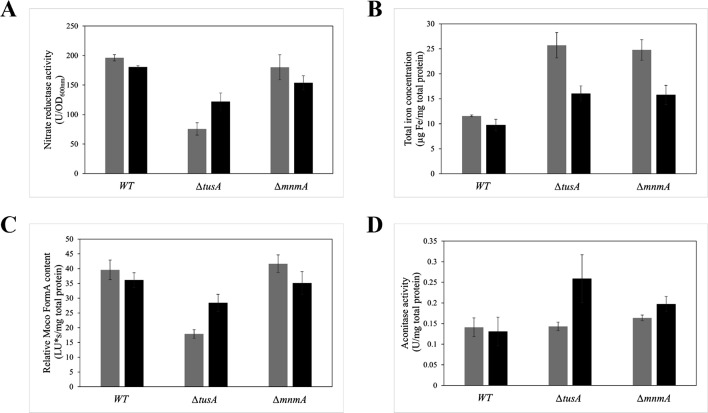
Enzyme activities and iron content of *E. coli* strains. Strains BW25113, ∆*tusA,* and ∆*mnmA*. (**A**) Nitrate reductase activity, (**B**) iron content, (**C**) Moco content, and (**D**) aconitase activity were measured in the respective strains (gray bars). Additionally, the effect of the expression of *fur* was tested after the introduction of a plasmid expressing *fur* (black bars). The strains were grown aerobically (**D**) or anaerobically (**A, B, C**) in LB medium supplemented with 15 mM potassium nitrate at 37°C for 7 h. The expression of *fur* was induced by adding 20 µM of Isopropyl β-d-1-thiogalactopyranoside (IPTG).

Additionally, we analyzed the overall iron content in the crude extract of strains BW25113, ∆*tusA*, and ∆*mnmA* (lacking tRNA-uridine 2-sulfurtransferase) after anaerobic growth for 7 h in Luria-Bertani (LB) dimedium supplemented with 15 mM potassium nitrate to confirm the Mössbauer data. The results in Fig. 3B show that under anaerobic growth conditions in the presence of nitrate, the overall iron content in the ∆*tusA* mutant strain was increased about two times compared to the BW25113 parental strain. To analyze whether the increased iron content was due to a defect in translational efficiency in the ∆*tusA* strain, we additionally analyzed a **∆***mnmA* strain for comparison. This strain also showed increased iron content to a similar extent observed for the **∆***tusA* strain (Fig. 3B).

Thus, we conclude that the higher iron levels are based on the reduced translation efficiency of a mRNA encoding a protein involved in iron import into the cell. One protein important to regulate the intracellular iron levels is Fur. Moreover, it was shown previously to be less abundant in a **∆***tusA* mutant strain (31). Therefore, we also analyzed the iron content in the **∆***tusA* and **∆***mnmA* mutant strains complemented with a plasmid expressing *fur* (Fig. 3B). Indeed, after the expression of *fur* in **∆***tusA*, the iron content decreased in this strain. Further, as reported previously, lowered nitrate reductase (NR) activity and lowered Moco content were present in the **∆***tusA* strain (Fig. 3A and C). However, after the expression of *fur* in this strain, increased NR activity and Moco levels were observed. On the other hand, these latter changes were not observed in the **∆***mnmA* strain and consequently were not rescued by the introduction of *fur*. This is likely based on the fact that TusA is the sulfur donor for Moco, and because MnmA has no role in Moco biosynthesis, no effect would be expected on the nitrate reductase activity or the Moco content observed (Fig. 3C).

Since the **∆***tus*A strain has a decreased [4Fe-4S] content (shown above), we tested the activity of aconitase in addition (Fig. 3D), under aerobic conditions. However, almost similar activities were obtained as compared to the BW25113 parental strain in both **∆***tusA* and **∆***mnmA* strains, while the activity in the **∆***tusA* strain increased after the expression of *fur* (Fig. 3D). Overall, when we complemented the ∆*tusA* and ∆*mnmA* strains with a plasmid expressing *fur*, the iron content dropped in these strains, showing that decreased Fur levels might be the reason for the increased iron content in these strains, the translation of which seems to be affected by the missing mnm^5^s^2^U34 thiomodifications in both strains.

### The translation of *fur* in different mutant strains

Previous results of Fur protein abundance determined by proteomics showed that Fur levels are reduced in a ∆*tusA* mutant (31). These reduced levels can be either based on a reduced transcription by Fe^2+^-Fur or a reduced translation by *rhyB*-Hfq or altered tRNA modification. Here, we analyzed the changed translation efficiency of *fur* mRNA in different mutant strains that affect tRNA thiomodifications.

We analyzed the translation efficiency of af*Fur*-EGFP fusion in mutant strains impaired in tRNA thiomodifications, like **∆***tusA*, **∆***mnmA*, **∆***iscS,* **∆***miaB*, **∆***ttcA,* and **∆***iscU,* and we analyzed the effect of tRNA thiomodifications on the translational efficiency of *fur*. Translational fusions were employed and included the T7 promoter and the *fur* coding sequence lacking the stop codon, allowing direct in-frame fusion to the coding sequence of EGFP as readout (41). The BW25113 parental strain and strains **∆***tusA*, **∆***mnmA*, **∆***iscS,* **∆***miaB*, **∆***ttcA,* and **∆***iscU* were transformed with the EGFP fusion reporter constructs in addition to the plasmids containing mCherry under the control of the T7 promoter without a gene fusion. The mCherry fluorescence thereby serves as a control to assay for changes in translational efficiency caused by the mutation of the respective regulatory gene on the reporter itself.

**Fig 4:**
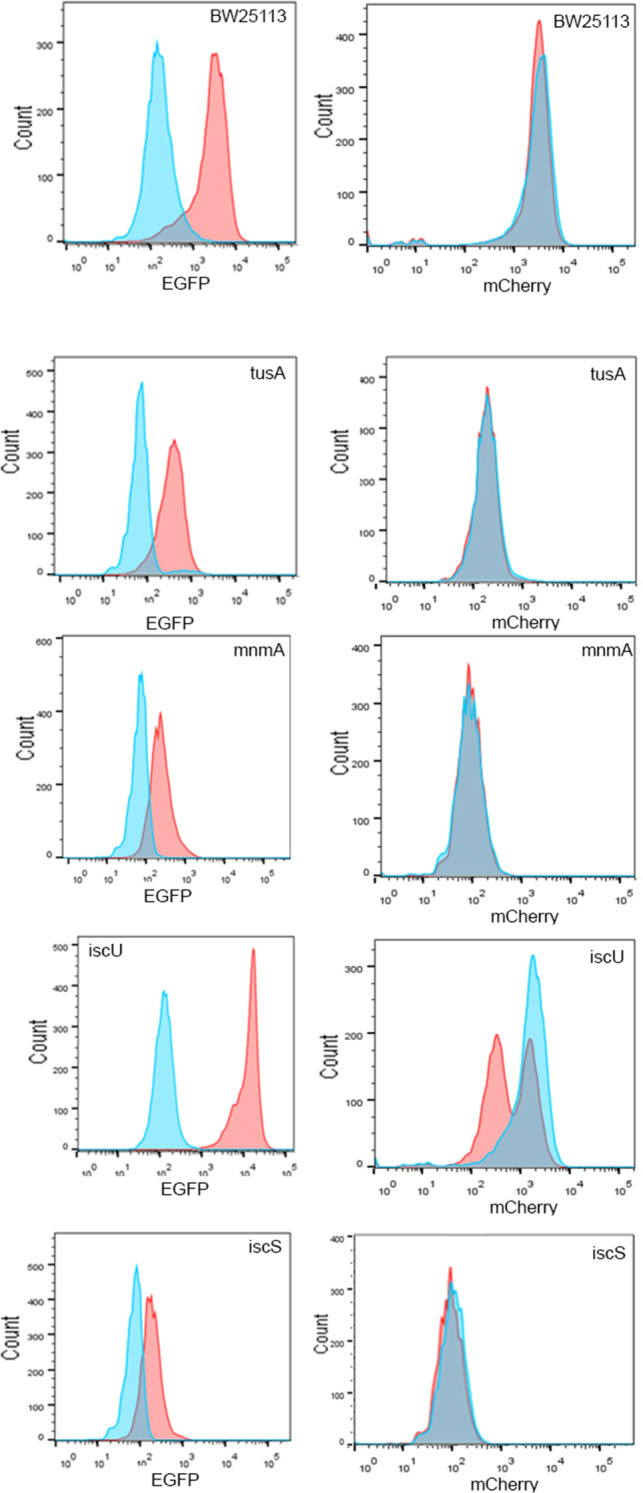
Fluorescence quantification of *fur*-EGFP translational gene fusions expressed in strains with deletion of genes responsible for mnm^5^s^2^U34 tRNA modifications. Translation efficiencies of *fur-EGFP* were analyzed in the BW25113 (DE3) parental strain and the strains **∆***tusA*, **∆***mnmA*, **∆***iscU*, **∆***iscS*, **∆***ttcA*, and ∆*miaB* transformed with either the plasmids *fur*-EGFP-pACYCDuet-1/mCherry-pCDFDuet-1 or the empty EGFP-pACYCDuet-1/mCherry-pCDFDuet-1 as a reference. Blue peaks show the fluorescence of the empty EGFP-pACYCDuet-1/mCherry-pCDFDuet-1 plasmid reference, and red peaks show the fluorescence from the *fur*-EGFP-pACYCDuet-1/mCherry-pCDFDuet-1 fusions. (**A**) The fluorescence of EGFP was recorded at an excitation of 488 nm and an emission of 507 nm. (**B**) The fluorescence of mCherry was recorded at an excitation of 587 nm and an emission of 610 nm. The distributions shown are taken from single experiments and are representative of three independent experiments.

The EGFP and mCherry fluorescence levels were measured by flow cytometry and were compared in the different mutant strains after 5 h of growth when cells were in the logarithmic growth phase (Fig. 4).

In BW25113 wild-type and each mutant strain, the EGFP-fusion and mCherry fluorescence were determined and compared to the fluorescence determined in the same mutant strains that contained mCherry and EGFP without gene fusion. Figure 4 shows the flow cytometry fluorescence obtained from the *fur*-EGFP fusion measured in strains BW25113, **∆***tusA* and **∆***mnmA*, **∆***iscS* **∆***miaB*, **∆***ttcA,* and **∆***iscU*. The fluorescence obtained for the mCherry control in the different mutant strains did not differ when the strains containing the *fur*-EGFP reporter fusion were compared with the ones containing only the EGFP reporter plasmid. This shows that, generally, the translational efficiency of the fluorescence reporter plasmid was not altered by deficiencies in tRNA thiolation. In comparison, the *fu*r-EGFP fusion showed a lower fluorescence in the BW25113 parental compared to the EGFP alone, correlating with lower levels of EGFP expression in the *fur* fusion protein. Higher fluorescence levels of the *fur*-EGFP fusion were only observed in the ∆*iscU* mutant strain, but not in the **∆***tusA*, **∆***mnm*A, or **∆***iscS* strains. In the strains with impaired mnm^5^s^2^U34 tRNA modifications, the fluorescence levels of EGFP and *fur*-EGFP were lowered as in the BW25113 parental strain. This shows that the absence of mnm^5^s^2^U34 tRNA modifications has a negative effect on the translational efficiency of Fur. Thus, an impairment in mnm^5^s^2^U34 tRNA modifications results in decreased cellular levels of Fur.

In the **∆***iscU* deletion strain, an even slightly higher fluorescence was obtained for the *fur*-EGFP fusion as compared to the fluorescence obtained in BW25113. In the **∆***tusA* mutant, the fluorescence of the *fur*-EGFP fusion in comparison to the EGFP fluorescence alone was reduced but was still increased when the fusion protein was expressed. Even a lowered level in the *fur*-EGFP fluorescence was obtained in the **∆***mnmA* and **∆***iscS* mutants. However, in these strains, the fluorescence was also not completely reduced to the fluorescence levels obtained for EGFP alone. This might indicate that tRNA modifications other than the mnm^5^s^2^U34 modification might influence the translation efficiency of *fur* translation. Therefore, we also analyzed **∆***miaB* and **∆***ttcA* strains, which lack gene products involved in s^2^C32 and ms^2^i^6^A37 tRNA modifications, respectively. The results showed that a similarly decreased level of *fur-*EGFP fusion was obtained, indicating that these tRNA modifications have a positive effect on the translation of Fur. These two tRNA modifications are Fe-S cluster-dependent, and because Fe-S cluster levels are impaired in the **∆***tusA* mutant, these tRNA modifications might also be affected, with the consequence of a negative impact on the translation efficiency of target genes, as shown in the example of *fur* in our studies. The complementation of the **∆***tusA* mutant strain with a plasmid expressing *fur* from an IPTG-inducible promoter did indeed rescue the **∆***tusA* phenotype and consequently resulted in reduced iron levels. This shows that the iron accumulation observed in the **∆***tusA* mutant strain is likely a result of a reduced translation of *fur* mRNA based on the lack of multiple tRNA thiomodifications.

### Immunodetection of IscS and SufS in different *E. coli* strains

**Fig 5:**
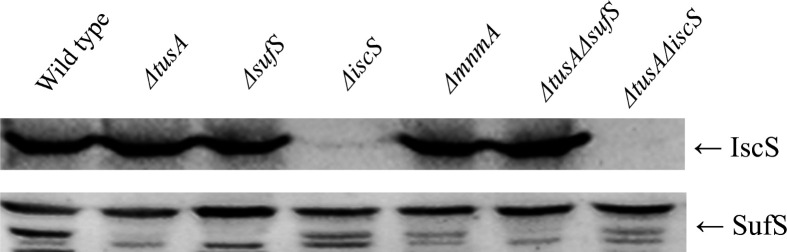
Quantification of IscS and SufS levels by immunodetection in different *E. coli* deletion strains. Western blots of extracts derived from *E. coli* strains BW25113 (parental strain), *∆tusA, ∆sufS, ∆iscS, ∆mnmA, ∆tusA/∆sufS,* or *∆tusA/∆iscS* were detected using IscS-specific antiserum (1:5,000) or a SufS-specific antiserum (1:5,000). The prestained protein molecular weight marker (Thermo Scientific; 20–120 kDa) was used as a reference.

Since a **∆***tusA* mutant shows reduced [4Fe-4S] cluster levels, increased iron levels, and reduced Fur levels, we wanted to determine any effect of the mutation on the abundance of IscS and SufS, two proteins central to Fe-S cluster assembly in *E. coli*. Immunodetection of SufS and IscS in strains ∆*tusA* and ∆*mnmA* that were cultivated for 7 h under anaerobic conditions in the presence of 15 mM potassium nitrate and target proteins was visualized by enhanced chemiluminescence (Fig. 5).

### Proteomic analysis of *ΔtusA* and *ΔmnmA* in the presence or absence of iron

To investigate the influence of iron and the *ΔtusA* and *ΔmnmA* deletion mutations on the overall protein abundance, a detailed proteomic analysis was performed. Cells of both strains and the corresponding BW25113 parental strain were cultivated in a medium with KNO_3_ as an electron acceptor, and iron was removed from the medium by the addition of 150 M 2,2-DIP.

The data of the western blot were confirmed by proteomics data (Table S1). Proteomic data showed that, both in ***∆**tusA* and ***∆**mnmA* mutants, the relative abundance of proteins of the Suf system was reduced, while, as expected, an increase in abundance was observed when dipyridyl as an iron chelator was added (Table S1). Data are available via ProteomeXchange with identifier PXD052252. This shows that the Suf system in these strains is mainly responsive to iron concentration.

In contrast, the western blot analysis showed that the proteins of the Isc system mainly essentially remained constant under these conditions in the tested strains. Nevertheless, as a lower Fe-S cluster level in the mutants was determined using Mössbauer spectroscopy, we also analyzed the abundance of the CyaY protein, which is a negative regulator for the activity of the IscS protein. Here, we obtained an increased abundance of the CyaY protein in the ***∆****tusA* mutant, which was decreased in the presence of dipyridyl. However, no changes were obtained in the **∆***mnmA* mutant, implying that the altered changes in the CyaY abundance were not because of the altered translation levels of CyaY.

### Test of the activity of IscR by analysis of the transcription of an *iscR-lacZ* fusion

**Fig 6:**
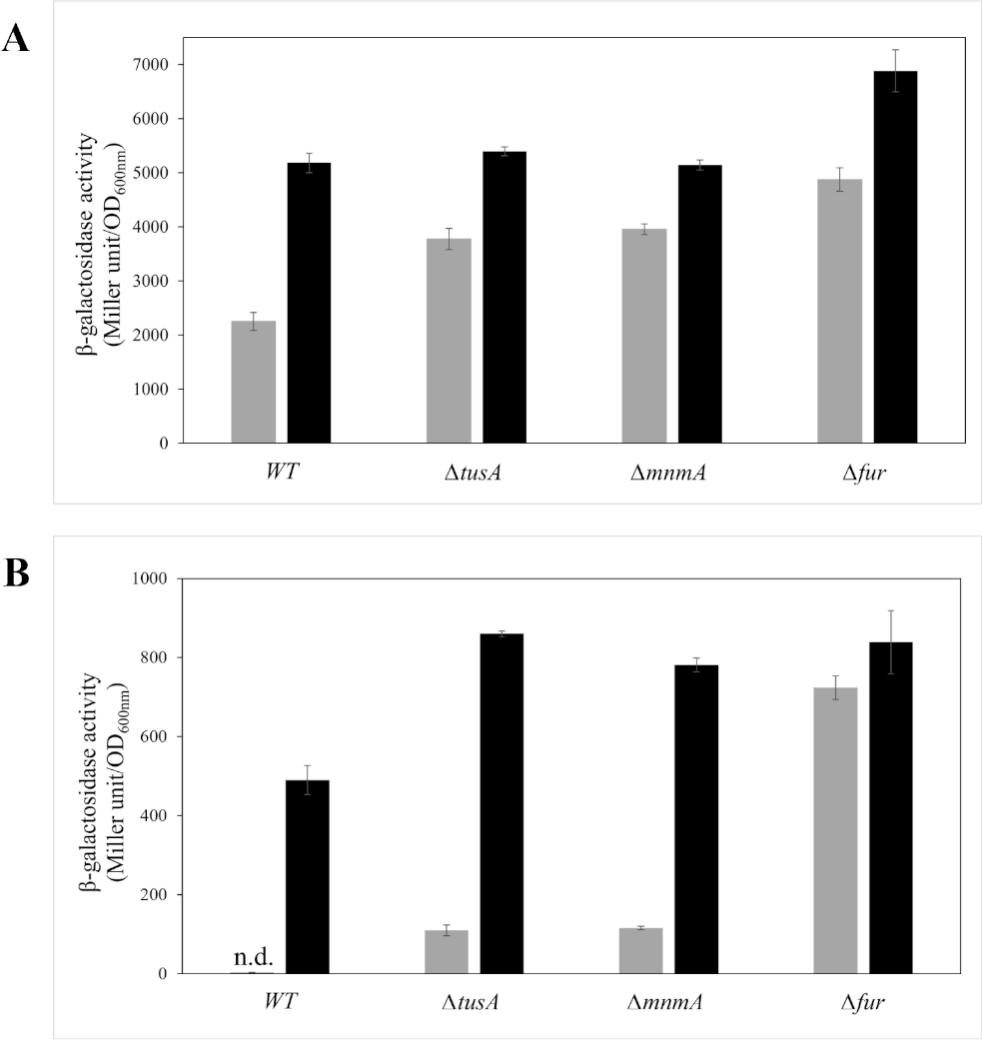
Expression of iscR-lacZ and ryhB-lacZ fusions in different *E. coli* strains. The expression of iscR-lacZ (**A**) and ryhB-lacZ (**B**) fusions was determined as β-galactosidase activity in the *E. coli* BW25113 parental strain, ΔtusA, ΔmnmA, and Δfur strains. Cells were grown anaerobically in the LB medium including 15 mM potassium nitrate with (black bars) or without (light gray bars) the addition of 100 µM of dipyridyl at 37°C for 7 h. The activity is calculated in Miller units and related to OD_600 nm_ from three independent measurements.

A reduction in Fe-S cluster levels should, however, lead to an increase in the expression of the *isc* operon, since IscR represses the transcription of *iscSUA* only in its Fe-S cluster-bound form. Indeed, by analyzing the transcription of an *iscR-lacZ* fusion in the **∆***tusA* mutant, an increase in the β-galactosidase levels was observed (Fig. 6A). However, the *isc* operon is additionally regulated by the small RNA *rhyB*, which leads to the degradation of the *iscSUA* mRNA. By analyzing the transcription of a *ryhB-lacZ* fusion, the abundance of *rhyB* was shown to be increased in the **∆***tusA* mutant and was even further increased by the addition of dipyridyl (Fig. 6B). Thus, while the expression of the *isc* operon was increased, based on lower Fe-S cluster levels detected in the **∆***tusA* mutant, the overall Isc protein concentration was not increased, because increased *ryhB* levels are known to cause degradation of *isc* operon mRNA.

Therefore, additional unidentified factors, other than IscS abundance, must lead to the observed decrease in Fe-S cluster levels in the **∆***tusA* mutant.

### Analysis of the transcription of *cyaY* by a *cyaY-lacZ* fusion

One factor that influences Fe-S cluster assembly might be the CyaY protein, as indicated above. The CyaY protein was reported to inhibit the activity of the IscS protein, thereby leading to a reduction in the cellular Fe-S cluster production. As shown above, the levels of the CyaY protein were indeed increased in the **∆***tusA* mutant strain. We, therefore, wanted to confirm the proteomics data by also analyzing whether an effect on gene expression could be observed. We analyzed the expression of a *cyaY-lacZ* fusion in the **∆***tus*A mutant and determined an approximately twofold increase compared to the expression level in the parental strain BW25113 (Fig. S1; supplementary material). The effect was only slightly reduced by the addition of dipyridyl, indicating that the expression of CyaY is likely not regulated by the cellular iron concentration, as suggested previously (42).

### L-Cysteine desulfurase activities

**Fig 7:**
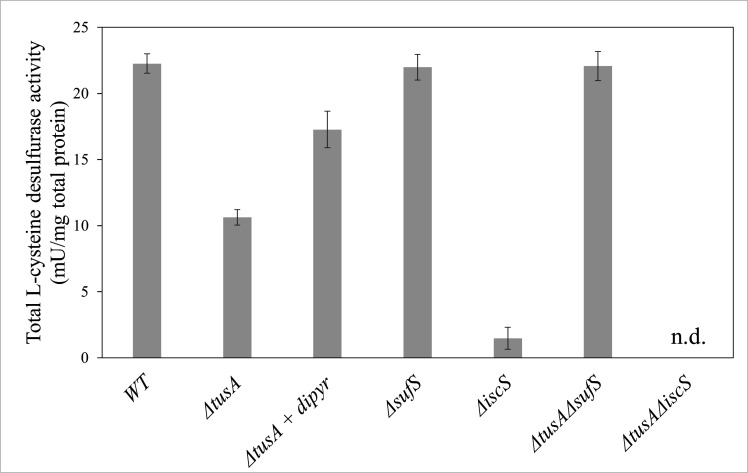
L-Cysteine desulfurase activities in different *E. coli* strains. L-Cysteine desulfurase activity in *E. coli* BW25113 parental strain, ΔtusA, ΔsufS, ΔiscS, ΔtusA/ΔsufS, and ΔtusA/ΔiscS mutants after anaerobic growth in LB supplemented with 15 mM potassium nitrate at 37°C for 7 h is shown. The ΔtusA mutant was grown with or without 100 µM of dipyridyl. Error bars, standard deviation from at least three different measurements; n.d., no activity determined.

The results shown above revealed that the absence of TusA resulted in decreased cellular Fe-S cluster levels, reduced SufS abundance, and increased CyaY levels, effects that together influence the cellular L-cysteine desulfurase activities of IscS and SufS. We, therefore, measured the cellular L-cysteine desulfurase activities in **∆***tus*A, **∆***sufS* (measuring mainly IscS activity), **∆***iscS* (measuring mainly the activity of SufS), **∆***tus*A**∆***sufS* (measuring the effect of the absence of TusA on IscS activity), and **∆***tusA***∆***iscS* mutants (measuring the effect of the absence of TusA on SufS activity). The results depicted in Fig. 7 show that the L-cysteine desulfurase activity was decreased by half in the **∆***tusA* mutant, which is consistent with the fact that the enhancing effect of TusA on IscS activity is absent and that SufS alone cannot compensate for this difference. By comparison, the measured activity for the **∆***sufS* mutant and the **∆***tusA***∆***sufS* mutants was comparable. In these strains, mainly the activity of IscS was measured, which remained unchanged, because of the absence of the enhancing effect of TusA and the presence of increased amounts of the inhibitor CyaY. In the **∆***tus*A**∆***iscS* mutant, no cysteine desulfurase activity was detected, due to the absence of SufS in this strain, which was not synthesized under high iron concentrations.

In the **∆***tusA* mutant, L-cysteine desulfurase showed a twofold reduction in activity, consistent with a decreased level of Fe-S clusters in this strain, and in the presence of dipyridyl, the situation was reverted, showing that the reduced L-cysteine desulfurase activity is due to the increased iron concentration in the **∆***tusA* strain. Indeed, this also results in an increased CyaY abundance, which inhibits the activity of IscS.

## DISCUSSION

In this report, we show that the ***∆****tus*A mutant strain has pleiotropic defects, not only on Moco biosynthesis and mnm^5^s^2^U34 tRNA modifications but also on the cellular Fe homeostasis and Fe-S cluster assembly. We were able to reveal that the increased iron levels in the ***∆****tu*sA mutant strain are mainly due to the reduced translation of *fur* mRNA, the main regulator of Fe homeostasis in *E. coli*. The lowered Fe-S cluster levels are additionally caused by a reduced expression of the *suf* operon under increased iron concentrations, as the operon is only expressed under iron-limiting conditions in *E. coli* (27). Fur controls the intracellular iron levels and is part of a complex regulatory network controlling the expression of more than 100 genes in *E. coli* (33), and it binds Fe^2+^ to repress the genes involved in iron uptake (43). Since the levels of Fur were lowered in the ***∆****tusA* mutant, Fe^2+^ uptake was no longer repressed, and consequently, Fe^2+^ accumulated in the ***∆****tusA* mutant strain, as was shown by whole-cell Mössbauer spectroscopy. Fur has further been shown to be involved in the regulation of Fe-S cluster biosynthesis by negatively regulating *sufABCDSE* operon expression and by the repression of the small regulatory RNA *ryhB* (44). It also represses the transcription of FNR and, thereby, influences the expression of most molybdoenzymes in *E. coli*, which was elevated in the ***∆****tusA* strain (45).

In this report, we investigated the contribution of TusA to Fe-S cluster and Moco biosynthesis under conditions that simultaneously require sulfur for both pathways. Previously, it was shown that Moco biosynthesis depends on Fe-S cluster biosynthesis for the activity of the MoaA protein, a two [4Fe-4S] cluster-containing protein belonging to the family of radical SAM enzymes (46, 47). MoaA, together with MoaC, catalyzes the first step of Moco biosynthesis, the conversion of 5′-GTP to cPMP (48–50). Further, many molybdoenzymes, like nitrate reductase, bind numerous Fe-S clusters, which are needed for intramolecular electron transfer reactions (51). Mössbauer spectroscopy revealed that Fe-S cluster species are reduced two- to threefold in single *tusA* mutants. Under aerobic conditions, in the absence of TusA, the low-spin Fe^2+^ species were largely reduced, with a simultaneous increase in high-spin ferrous iron species. In contrast, only a decrease in the Fe^3+^-based Fe-S centers was observed. Surprisingly, the accumulation of Fe^2+^ did not result in an increase in ROS in these cells (data not shown). The accumulation of iron in the ***∆****tusA* strain might also be the consequence of a defect in Fe-S cluster assembly. Here, we could demonstrate that the levels of SufS were decreased in the ***∆****tusA* strains, also under anaerobic conditions. Since no increase in ROS production was observed by the increased Fe^2+^ levels, this raises the question of where the ferrous iron species are stored. We could not, however, detect an accumulation of Fe^2+^ in ferritin (FtnA) or bacterioferritin (Bfr) (data not shown) (35). Nevertheless, Fe^2+^ might not be present in a free state in the cell, and cellular ROS production might be prevented by binding the iron to glutathione or other small molecules in the cell (52). Further, an increase in Fe levels was also observed for the ***∆****mnmA* strain. This would rather suggest that a lack of tRNA mnm^5^s^2^U34 thiomodifications influences the translation of a protein involved in Fe import. We were able to determine that this protein is Fur, the translation of which was reduced in all strains lacking genes required for thiomodifications of tRNA.

The role of TusA in the assembly of Fe-S clusters is a novel function for this protein. So far, the interaction sites of IscU, ThiI, TusA, IscX, Fdx, and CyaY have been mapped on IscS (22). Previous studies predicted that these proteins bind to IscS only individually and not together (19). Thus, under our conditions of investigation, the absence of TusA should rather have a positive effect on Fe-S cluster biosynthesis, since more IscS would be available to interact with IscU, CyaY, and Fdx for Fe-S cluster assembly. Furthermore, TusA has been described to enhance the activity of IscS, increasing its activity about twofold (31). When TusA is absent, the activity of IscS is consequently not increased. Further, the abundance of CyaY was slightly increased in the **∆***tusA* mutant, as revealed both by transcriptional studies and on the protein level as shown by proteomics analyses. Since CyaY acts as a repressor of IscS activity, the activity of the protein is consequently reduced, leading to lowered Fe-S cluster formation. This behavior cannot be compensated for by SufS in the **∆***tusA* mutant, since SufS is nearly absent, due to the increased iron levels in this strain. In the cellular context, the absence of TusA coincides with a lack of mnm^5^s^2^U34 thiomodifications that result in altered translation efficiencies of numerous proteins, among which Fur seems to be a major target. Lowered cellular amounts of Fur result in an increase in cellular Fe levels and a decrease in Fe-S cluster content, consequently resulting in pleiotropic effects due to altered Fe homeostasis. Our studies conclusively show that the pleiotropic effects of a *tusA* mutation might be caused by changes in the Fe homeostasis of the cell, leading to major differences in gene regulation, including altering the levels of CyaY, which then reduces the activity of IscS and leads to decreased cellular Fe-S cluster production.

## MATERIALS AND METHODS

### Media and growth conditions

The plasmids and strains used in this study are listed in Table 2. *E. coli* cultures were grown in the LB medium under aerobic or anaerobic conditions supplemented with 100 µM FeCl_3_ (for Mössbauer experiments) and at 37°C. Where indicated, 15 mM potassium nitrate was additionally added to the medium during growth.

**TABLE 2:** *E. coli* strains and plasmids used in this study

Plasmid or strain	Genotype or relevant characteristics	Reference
*pMB30 (His)_6_-Fur*	*fur* coding region cloned into NdeI/BamHI sites of pET15b, Amp^r^	This work
*pEGFP*	*egfp* coding region cloned into pACYC-Duet1, Cam^r^	(41)
*pFur-EGFP*	*fur* coding region cloned into pEGFP, Cam^r^	This work
*pmCherry*	*mCherry* coding region cloned into pET11b, Spec^r^	(41)
*piscR-lacZ*	Gene region 200–21 bp upstream of *iscR* transcriptional start cloned into EcoRI/BamHI sites of pGE593, Amp^r^	This work
*pcyaY-lacZ*	*cyaY* promotor region cloned into EcoRI/BamHI sites of pGE593, Amp^r^	This work
*pryhB-lacZ (pMB34*)	*ryhB* promotor region cloned into EcoRI/BamHI sites of pGE593, Amp^r^	This work
*ΔtusA* strain	BW25113 derivate, *ΔtusA::kan*	(53)
*ΔmnmA* strain	BW25113 derivate, *ΔmnmA::kan*	(53)
*ΔttcA* strain	BW25113 derivate, *ΔttcA::kan*	(53)
*ΔmiaB* strain	BW25113 derivate, *ΔmiaB::kan*	(53)
*ΔiscU* strain	BW25113 derivate, *ΔiscU:::kan*	(53)
*ΔiscS* strain	BW25113 derivate, *ΔiscS::kan*	(53)
*ΔsufS* strain	BW25113 derivate, *ΔsufS::kan*	(53)
*ΔtusAΔsufS* strain	BW25113 derivate, *ΔtusAΔsufS::kan*	(53)
*ΔtusAΔiscS* strain	BW25113 derivate, *ΔtusAΔiscS::kan*	(53)
*Δfur* strain	BW25113 derivate, *Δfur::kan*	(53)

Mössbauer samples were prepared by the addition of 100-µM ^57^Fe-labeled ferric citrate to the culture medium. ^57^Fe solutions were prepared from 33.5 mg of ^57^Fe dissolved in 1 mL of 8 M HCl at room temperature for 12 h. The volume was adjusted to 5 mL with water, and 0.68 mM of trisodium citrate dihydrate was added. The solution was neutralized with ammonia to a pH of 5–6, forming a working ammonium ferric citrate stock solution. This stock solution was added to the LB medium (pH 7.2) to a final concentration of 100 µM ^57^Fe.

Unless noted otherwise, the temperatures used to grow the strains were either 30°C or 37°C.

BW25113 and the isogenic mutant strains ∆*tusA,* ∆*sufS,* ∆*iscS,* ∆*ttcA,* ∆*miaB,* ∆*fur,* ∆*iscU,* and ∆*mnmA* (Keio collection) were obtained from the National BioResource Project (National Institute of Genomics, Japan) (53).

Double mutants were constructed by first deleting the kanamycin resistance cassette using the plasmid pCP20 and subsequently deleting the second gene of interest using the plasmid pKD46. Amplified DNA fragments were used by the recombinase (expressed by pKD46). Each strain was verified by colony PCR.

The wild-type BW25113 and the deletion mutants were transformed with pET15b plasmid for *fur* expression (Amp^R^). The strains and mutants used to overexpress *fur* were DE3-competent cells. DE3 (*lacZ* promoter + T7 polymerase gene) was inserted using the λDE3 Lysogenization Kit (Novagen, Sigma-Aldrich). The overexpression of *fur* was started by induction with 20 µM IPTG.

### Protein concentration quantification

Protein concentrations were determined using the Bradford Reagent Coomassie Plus Protein Assay Reagent (Thermo) with bovine serum albumin as a standard following the manufacturer’s instructions.

### Aconitase activity assay

Aconitase activity was assessed in cell extracts after 7 h of aerobic growth in the LB medium. Cells of the *E. coli* parental strain BW25113 and the deletion mutants were harvested and washed with Tris/HCl 50 mM pH 7.5. For aconitase activities, cells were lysed by sonication in 50 mM Tris-HCl and 150 mM NaCl (pH 8.0). Aconitase activity was determined in a coupled enzymatic assay monitoring NADPH production at 340 nm from the oxidation of isocitrate produced by isocitrate dehydrogenase. A 50 µL cell lysate was incubated for 5 min in 450-µL 50-mM Tris-HCl, 50-mM NaCl, 5-mM MgCl_2_, 0.5-mM NADP^+^, and 0.05-U isocitrate dehydrogenase (pH 8.0). The reaction was started by the addition of 500-µL 2.5-mM cis-aconitate in the same buffer. One unit is defined as 1-µmol NADPH formed in 1 min. Aconitase activity was normalized to the total protein concentration used in the assay.

### Nitrate reductase activity assay

The activity of nitrate reductase was measured in crude extracts obtained from *E. coli* strains BW25113 wild-type, ∆*tusA,* and ∆*mnmA* after anaerobic growth for 7 h in the presence of 15-mM potassium nitrate. Cells were harvested in the stationary growth phase by centrifugation and resuspended in 50 mM Tris-HCl (pH 7.5). Cell lysates were obtained by sonication, transferred into an anaerobic chamber, and incubated at 4°C for at least 3 h. Fifty microliters of each cell lysate was analyzed for nitrate reductase in a volume of 4 mL containing 0.3-mM benzyl viologen and 10-mM KNO_3_ in 20-mM Tris-HCl (pH 6.8). The assay was initiated by injecting sodium dithionite into the anaerobic reaction mixture until OD_600_ of 0.8–0.9 for reduced benzyl viologen was reached. After the addition of crude extract, the oxidation of benzyl viologen was recorded at 600 nm for 30 sec. The activity was calculated using the equation *U* = 0.5× (∆Abs_600_/min)/ε_600_(benzyl viologen)/*V*, using the extinction coefficient for benzyl viologen of 7.4 mmol^−1^ × cm^−1^. One unit is defined as the oxidation of 1 µmol reduced benzyl viologen per minute. The activity was normalized to the OD_600_ of the cells before harvesting.

### Moco FormA quantification

The *E. coli* parental strain BW25113 and the deletion mutants were anaerobically cultivated at 37°C for 7 h in the LB medium with the addition of 15 mM of potassium nitrate. After harvesting and washing with 50 mM Tris-HCl (pH 7.5) buffer, cells were suspended in the same buffer and sonicated. The cell debris and unbroken cells were removed by centrifugation at 13,200 × *g* for 20 min at 4°C. An aliquot of 400 µL of supernatant was incubated with 50 µL of KI-HCl and 150 µL of KI and heated at 95°C for 30 min (in a brown Eppendorf tube), followed by an overnight incubation in the dark. The samples were then centrifuged at 16,200 × *g* for 30 min; 400 µL of treated cell supernatant was transferred in a new test tube. One hundred microliters of ascorbic acid 1%, 200 µL of Tris-HCl 1 M, 30 µL 1 M MgCl_2_, and 2 µL of Fast AP were added to the tube. The samples were kept in the dark for 2 h at room temperature. Moco FormA was then extracted by ion-exchange chromatography with acetic acid 10 mM. The four elution fractions obtained were loaded onto an HPLC (Agilent Technologies 1260 Infinity) and separated on a C18 column to quantify the FormA of Moco (used buffer ammonium acetate 5 mM and methanol 80:20).

The amount of Form A obtained from the measurement was then normalized to the total protein concentration used in the assay.

### Total iron concentration quantification

For metal analysis of crude extract, the strains *E. coli* BW25113 parental strain, ∆*tusA,* and ∆*mnmA* cells were grown for 7 h anaerobically in 50 mL LB supplemented with 15 mM potassium nitrate at 37°C. Cultures were harvested; cells were washed three times with 10 mL 50 mM Tris-HCl (pH 7.5), suspended in 3 mL of the same buffer, and sonicated. Two milliliters of disrupted cells were centrifuged at 18,000 × *g* for 30 min. Metal analysis was performed using a PerkinElmer (Waltham, MA) Life Sciences Optima 2100DV inductively coupled plasma optical emission spectrometer as described earlier (54). As a reference, the multi-element standard solutions XVI (Merck) was used.

### Flow cytometry of EGFP fusion proteins

The translation levels of the EGFP fusion proteins were measured by flow cytometry as described previously (41). The *E. coli* BW25113 parental strain and the respective mutant strains were DE3-lysogenized and transformed with *fur*-EGFP-pACYCDuet-1 and mCherry-pCDFDuet-1 vectors. The expression of mCherry served as an internal control for translation. In addition, the strains were also transformed with corresponding empty EGFP-pACYCDuet-1 and mCherry-pCDFDuet-1 vectors as an additional internal control. Precultures of the *E. coli* strains were grown in a M9 minimal medium overnight at 37°C with 200 rpm. The next day, the cells were transferred to 50 mL of LB at a starting OD_600 nm_ of 0.05, and the cells were grown at 37°C with 180 rpm for 5 h. The expression of fusion proteins was induced with 100 mM IPTG at time point 0 h. After 5 h of growth, cell cultures were transferred to 50-mL Falcon tubes, and the OD_600 nm_ was determined. The cell count for each sample was set to 10^8^ cells/mL for flow cytometry. Five hundred microliters of cells in 1× PBS was subjected to flow cytometry. Each sample was detected for EGFP and mCherry fluorescence signal using a fluorescence-activated cell sorting Melody system (Bioscience). In total, 10,000 cells were measured for each sample. EGFP was excited at 488 nm and mCherry at 587 nm. The fluorescence signal of EGFP was detected at 507 nm in the GFP channel, and the fluorescence of mCherry was detected at 610 nm in the mCherry channel.

### Immunodetection of IscS and SufS

*E. coli* BW25113 parental strain and all the other mutant strains were cultivated for 7 h under anaerobic conditions at 37°C in the presence of 15 mM potassium nitrate. After harvesting and washing with 50 mM Tris-HCl (pH 7.5) buffer, cells were suspended in the same buffer. Cells were lysed by sonification, and the cell debris was removed by centrifugation at 13,200 × *g* for 20 min. Protein concentration was quantified by the Bradford assay. Fifty micrograms of the cell extracts was separated by 12% (wt/vol acrylamide) SDS-PAGE and transferred to nitrocellulose or Polyvinylidene difluoride (PVDF) membranes (Amersham). The membrane was blocked with 5% (wt/vol) skim milk in Tris-buffered saline plus Tween (TBST) for 2 h at room temperature, rinsed with TBST, and incubated with chicken anti-SufS serum or rabbit anti-IscS serum overnight at 4°C. The blot was washed with TBST and incubated with horseradish peroxidase-conjugated goat anti-chicken antibodies (Abcam) or goat anti-rabbit secondary antibodies (Thermo Scientific). Target proteins were visualized by enhanced chemiluminescence.

### β-Galactosidase activity measurements

*E. coli* BW25113 parental strain and the *E. coli* mutant were transformed with the gene promotor of interest fused to the *lacZ* reporter gene and were cultivated anaerobically at 37°C for 7 h with the addition of 15 mM of potassium nitrate and in the presence or absence of 100 µM of dipyridyl, according to the experimental conditions. The β-galactosidase activities were measured using the SDS-chloroform method.

Bacterial cells were added to 500 µL of Buffer Z (60 mM Na_2_HPO_4_, 40 mM NaH_2_PO_4_, 10 mM KCl, and 1 mM MgSO_4_ pH 8), 25 µL of SDS 0.1%, and 50 µL of chloroform. The samples were incubated at 28°C for 5 min. The reaction was started by adding 100 µL of o-nitrophenol-β-D-galactopyranoside (4 mg/mL) and incubated at 28°C. The reaction was stopped by adding 250 µL of Na_2_CO_3_ 1 M. The produced o-nitrophenol amount was measured at 420 nm, corrected for light scattering at 550 nm, and normalized to their optical density at 600 nm, the reaction time, and the volume of the cells (Miller units). For each assay, BW25113 cells transformed with empty pGE593 vector were used as blank and subtracted from each value.

### Quantification of the total L-cysteine desulfurase activity from cell extracts

The *E. coli* BW25113 parental strain and other mutant strains were grown anaerobically in the LB medium for 7 h, supplemented with 15 mM of potassium nitrate with or without 100 µM of 2,2′-dipyridyl according to the experimental conditions. The cells were harvested, washed with Tris/HCl 50 mM pH 7.5, and used in the assay. The total L-cysteine desulfurase activity from the cell crude extracts was quantified using the methylene blue assay by following published procedures (6).

Cell extracts were incubated with 1 mM Dithiothreitol (DTT) and 1 mM L-cysteine for 10 min at 30°C. The reaction was stopped, and each product was quantified by using a standard sulfide calibration curve (0–200 µM sulfide). One unit is defined as the amount of enzyme producing 1 µmol of sulfide/minute.

### Proteomic analysis

Bacteria were grown anaerobically in the LB medium until the mid-log phase. After washing in 50 mM Tris-HCl, pH 8.0, cells were pelleted, suspended in the previously mentioned buffer, and sonicated. One hundred micrograms of cell extract was mixed with 8 M urea in 10 mM Tris-HCl, pH 8.0, and loaded on filter columns (Microcon-30 kDa Centrifugal Filter Unit with Ultracel-30 membrane; Merck-Millipore). Columns were washed with 8 M urea in 10 mM Tris-HCl, pH 8.0, reduced using 10 mM DTT in 8 M urea, and alkylated using 27 mM iodoacetamide in 10 mM Tris-HCl, pH 8.0. Afterward, columns were mixed at 600 rpm in a thermomixer for 1 min and incubated without mixing for a further 5 min. 8 M urea in 10 mM Tris-HCl, pH 8.0, was added to each column and centrifuged. After this step, 14-h digestion with trypsin was performed. Reactions were stopped by the addition of 10% Trifluoroacetic acid (TFA). Peptides were purified on C18 SepPack columns (Teknokroma), eluted with 800 µL 60% acetonitrile (ACN) and 0.1% TFA, and dried in a speed vacuum concentrator. Dried peptides were resuspended in MS loading buffer (3% ACN, 0.1% FA) and measured with Q Exactive HF (Thermo Fisher Scientific, Hennigsdorf, Germany) coupled to a reverse-phase nano liquid chromatography Acquity UPLC M-Class system (Waters). The gradient ramped from 3.2% to 76% ACN. The gradient started from 3.2% ACN and increased to 7.2% ACN in the next 20 min, then to 24.8% ACN over 70 min and 35.2% ACN over the next 30 min, followed by a 5-min washout with 76% ACN. The MS was run using a data-dependent top-N method that fragmented the top 12 most intense ions per full scan. Full scans were acquired at a resolution of 120,000 with an AGC target of 3*e*6, maximum injection time of 50 ms, and scan range of 300–1,600 m/z. Each dd-MS2 scan was recorded in the profile mode at a resolution of 15,000 with an AGC target of 1*e*5, maximum injection time of 100 ms, isolation window of 1.2 m/z, and normalized collision energy of 27, and the dynamic exclusion lasted for 30 sec.

The mass spectrometry proteomics data have been deposited to the ProteomeXchange Consortium via the PRIDE [1] partner repository with the data set identifier PXD052252. The names are identical to the ones in this list in Table 1 in the SI.

Raw proteomics files were analyzed using MaxQuant software (Version 1.6.0.16) with Andromeda—an integrated peptide search engine. Peptides were identified by matching to the *E. coli* Uniprot protein sequence library using default orbitrap settings. Moreover, a maximum of two missed cleavages were allowed, and the threshold for peptide validation was set to 0.01 using a decoy database. In addition, methionine oxidation and N-terminal acetylation were considered as variable modifications while cysteine carbamidomethylation as a fixed modification. In the analysis, the following options were selected: “label-free quantification” and “match between runs,” and the minimum length of the peptide was set to at least seven amino acids. In the further analysis, only proteins with equal or more than two unique peptides were considered. Moreover, contaminants, i.e., keratins, were removed.

### Mössbauer spectroscopy

Mössbauer spectra were recorded in the constant acceleration mode with a conventional spectrometer and a multi-channel analyzer in the time-scale mode (WissEL GmbH). Mössbauer spectra were taken at 77 K using a bath cryostat cooled with liquid nitrogen (Oxford Instruments). After data transfer from the multi-channel analyzer to a PC, the public domain program Vinda (55) running on an Excel 2003 platform was used for data analysis. The Mössbauer spectra were analyzed by least-squared fits using Lorentzian line shapes with the line width at half maximum *Γ*. Isomer shifts *δ* are given relative to α-iron at room temperature.

## SUPPLEMENTAL MATERIAL

Supplemental material spectrum.00556-24-s0001.docx Fig. S1; Table S1.

ASM does not own the copyrights to Supplemental Material that may be linked to, or accessed through, an article. The authors have granted ASM a non-exclusive, world-wide license to publish the Supplemental Material files. Please contact the corresponding author directly for reuse.
