# Supplementary material for: TusA influences Fe-S cluster assembly and iron homeostasis in E. coli by reducing the translation efficiency of Fur
Source: Microbiol Spectr. 2024 Jun 25;12(8):e00556-24. doi: 10.1128/spectrum.00556-24 (PMC11302051; doi:10.1128/spectrum.00556-24)
Supplement: Supplemental material — Fig. S1; Table S1. [file spectrum.00556-24-s0001.docx]

# Supplementary material

TusA influences FeS cluster assembly and iron homeostasis in E. coli by reducing the translation efficiency of Fur

Paolo Olivieri^1^, Arkadiuz Zupok^1,^ Tugba Yildiz^1^, Jonathan Oltmanns ^2^, Angelika Lehmann^1^, Ewelina Sokolowska^3^, Aleksandra Skirycz^3,4^, Volker Schünemann ^2^and Silke Leimkühler^1^*

^1^Institute of Biochemistry and Biology, Department of Molecular Enzymology, University of Potsdam, D-14476 Potsdam, Germany; ^2^Department of Physics, University of Kaiserslautern-Landau, D-67663 Kaiserslautern, Germany; ^3^Max-Planck-Institute of Molecular Plant Physiology, Am Mühlenberg 2, 14476, Potsdam-Golm, Germany; ^4^Current address: Michigan State University, East Lansing, 48824, MI, USA,

Running title: TusA's role in Fe-S cluster assembly and Fe homeostasis

**^*^Corresponding Author**

Silke Leimkühler, Department of Molecular Enzymology, Institute of Biochemistry and Biology, University of Potsdam, Karl-Liebknecht-Str. 24-25, 14476 Potsdam, Germany; Tel.: +49-331-977-5603; Fax: +49-331-977-5128; E-mail: sleim@uni-potsdam.de

**Table S1: Proteomic data in *ΔtusA* and *ΔmnmA* in LB ± 150 µM dipyridyl against BW25113 parental strain.**

The cells were cultivated anaerobically in LB supplemented with 15 mM potassium nitrate with or without the addition of 150 µM 2,2’-dipyridyl for 7 h. After harvesting and washing with 50 mM Tris/HCl pH 8, the cells were used in proteomics analysis. The fold changes of each detected protein in the mutant strains, compared to the BW25113 wild type strain, are listed below.

|  | **WT** | ***ΔtusA*** | ***ΔtusA with dipyr*** | ***ΔmnmA*** |
| --- | --- | --- | --- | --- |
| **Moco Biosynthesis** |  |  |  |  |
| MoaA | 1 | 24.5859 | 6.6225 | 4.5807 |
| MoaB | 1 | 2.5185 | 1.5242 | 0.7431 |
| MoaC | 1 | 2.0589 | 1.2126 | 0.5577 |
| MoaD | 1 | 1.9615 | 1.1568 | 0.4921 |
| MoaE | 1 | 2.0536 | 1.3908 | 0.5791 |
| MoeA | 1 | 1.0249 | 1.2261 | 0.8210 |
| MoeB | 1 | 1.5769 | 1.5093 | 1.2096 |
| MogA | 1 | 1.4856 | 1.6501 | 1.2617 |
| ModA | 1 | 0.3159 | 0.2172 | 0.6077 |
| **ISC system** |  |  |  |  |
| IscU | 1 | 1.0723 | 1.1777 | 1.1150 |
| IscS | 1 | 0.8660 | 1.0438 | 0.9434 |
| IscR | 1 | 0.5894 | 2.1454 | 0.5918 |
| IscA | 1 | 1.9086 | 2.3469 | 1.5721 |
| IscX | 1 | 0.8965 | 0.9137 | 0.5847 |
| hscA | 1 | 1.0218 | 1.0015 | 1.0967 |
| hscB | 1 | 1.1134 | 1.7481 | 1.0194 |
| fdx | 1 | 1.1691 | 0.7752 | 1.1357 |
| **SUF system** |  |  |  |  |
| SufA | 1 | 0.2455 | 2.7093 | 0.2969 |
| SufB | 1 | 0.5849 | 4.7408 | 0.5367 |
| SufC | 1 | 0.5235 | 3.2937 | 0.5683 |
| SufD | 1 | 0.5435 | 5.2129 | 0.5971 |
| SufS | 1 | 0.4472 | 3.0706 | 0.4521 |
| SufE | 1 | 0.2040 | 5.5811 | 0.6741 |
| **Iron trafficking proteins** |  |  |  |  |
| FeoA | 1 | 0.0000 | 143.0062 | 3.2619 |
| FeoB | 1 | 1.2438 | 732.9978 | 13.8428 |
| FepA | no signal | no signal | no signal | no signal |
| FepB | 1 | 0.0000 | 3.1524 | 0.4146 |
| **Fe-S cluster carrier proteins** |  |  |  |  |
| ErpA | 1 | 1.6135 | 0.8425 | 1.3730 |
| NfuA | 1 | 0.8837 | 1.3272 | 0.9020 |
| **Format dehydrogenase** |  |  |  |  |
| FdnG | 1 | 0.7520 | 0.6153 | 1.7460 |
| FdnH | 1 | 5.1632 | 0.4170 | 2.2810 |
| **TMAO reductase** |  |  |  |  |
| TorA | no signal | no signal | no signal | no signal |
| TorC | no signal | no signal | no signal | no signal |
| **Periplasmic Nitrate reductase** |  |  |  |  |
| NapA | 1 | 0.6166 | 0.0056 | 2.3801 |
| **DMSO reductase** |  |  |  |  |
| DmsA | 1 | 0.4087 | 0.0000 | 0.7815 |
| DmsB | no signal | no signal | no signal | no signal |
| **Nitrate reductase** |  |  |  |  |
| NarG | 1 | 1.4468 | 0.3217 | 1.0419 |
| NarH | 1 | 1.4728 | 0.2041 | 0.8864 |
| NarI | 1 | 1.0473 | 0.2453 | 0.7825 |
| NarJ | 1 | 2.0813 | 1.1322 | 1.9449 |
| NarX | 1 | 1.2779 | 0.8637 | 1.3667 |
| NarK | 1 | 1.1251 | 0.8058 | 0.8611 |
| **Format dehydrogenase** |  |  |  |  |
| FdoG | 1 | 0.4727 | 0.3131 | 1.3411 |
| FdoH | 1 | 4.5437 | 0.3840 | 2.4305 |
| **Regulators in Moco and Fe-S cluster** |  |  |  |  |
| CsrA | 1 | 0.7765 | 1.2561 | 0.8424 |
| ArcA | 1 | 1.2357 | 0.9792 | 1.3657 |
| NarL | 1 | 1.1410 | 0.8186 | 1.1294 |
| NarP | 1 | 1.9758 | 0.9895 | 1.7129 |
| ModE | 1 | 0.9991 | 1.2586 | 0.8664 |
| RstA | 1 | 2.5731 | 1.7238 | 1.8066 |
| Fis | 1 | 9.2608 | 7.6832 | 6.6242 |
| IhfA | 1 | 0.7490 | 0.7398 | 0.7859 |
| IhfB | 1 | 0.8234 | 0.8301 | 0.9548 |
| TorR | no signal | no signal | no signal | no signal |
| RpoS | 1 | 0.3342 | 0.2576 | 1.7066 |
| CyaY | 1 | 1.2507 | 1.0248 | 0.9399 |
| **Format dehydrogenase** |  |  |  |  |
| FdhF | 1 | 3.2395 | 0.0000 | 1.7754 |
| **Xanthine dehydrogenase** |  |  |  |  |
| XdhA | no signal | no signal | no signal | no signal |
| XdhB | no signal | no signal | no signal | no signal |
| **xanthine/hypoxanthine oxidase** |  |  |  |  |
| XdhD | 1 | 4.5284 | 0.8026 | 2.2771 |
| **P-loop NTPase family protein Mrp** |  |  |  |  |
| Mrp | 1 | 1.7871 | 1.1649 | 1.4635 |
| **tRNA modification** |  |  |  |  |
| TusA | 1 | 0.0000 | 0.0000 | 1.7165 |
| TusB | no signal | no signal | no signal | no signal |
| TusC | no signal | no signal | no signal | no signal |
| TusD | no signal | no signal | no signal | no signal |
| TusE | 1 | 0.9370 | 1.9440 | 0.4690 |
| MnmA | 1 | 2.2251 | 2.7352 | 0.0000 |
| ThiI | 1 | 1.4132 | 1.4420 | 1.1959 |
| MiaB | 1 | 4.1044 | 0.8663 | 2.7256 |
| TtcA | 1 | 1.0167 | 0.8427 | 0.8126 |

The MS raw data are available vis the Proteome Exchange database Project No.: PXD 052252, the names are identical with the ones in this list.





**Figure S1: Expression of a cyaY-lacZ fusion in different E. coli strains**

The expression of a cyaY-lacZ fusions was measured as β-galactosidase activity in E. coli BW25113, ΔtusA ΔmnmA and Δfur strains. Cells were grown under anaerobic conditions in LB medium supplemented with 15 mM potassium nitrate at 37°C for 7h with (black bars) or without (light grey bars) the addition of 100 µM dipyridyl. The activity is calculated in Miller Units and related to OD_600_ nm from 3 independent measurements.
